# A Study on the Application Performance of High-Aspect-Ratio Nano-Ettringite in Photocurable Resin Composites

**DOI:** 10.3390/ma17143492

**Published:** 2024-07-14

**Authors:** Weihua Cao, Hong Zhu

**Affiliations:** School of Materials Science and Engineering, Nanjing Tech University, Nanjing 211816, China

**Keywords:** nano-ettringite, photocurable resin, viscosity, shrinkage, tensile strength

## Abstract

In this study, the impact of the addition of high-aspect-ratio nano-ettringite to photocurable epoxy acrylate resin was explored. The nano-ettringite samples were modified using γ-Aminopropyltriethoxysilane (KH-550) and γ-methacryloxypropyl trimethoxy silane (KH-570). Then, 3 wt% or 6 wt% KH-550-modified, KH-570-modified, and unmodified nano-ettringite samples were dispersed into resin via ultrasonic treatment in conjunction with mechanical stirring. The grafting effects of nano-ettringite onto KH-550 or KH-570 were analyzed through scanning electron microscopy (SEM), Fourier transform infrared (FTIR) spectroscopy, X-ray diffraction (XRD), and thermogravimetric (TG) analysis. The results demonstrate that KH-550 and KH-570 have been successfully grafted onto the surface of nano-ettringite. In addition, this study also focuses on the variations of composite materials in the viscosity, shrinkage, tensile strength, and elongation at break. The results indicate that increased dosages of unmodified, KH-550-modified, and KH-570-modified nano-ettringite led to increased viscosity of the composite while reducing shrinkage. At the same dosage, the photocurable resin containing KH-570-modified nano-ettringite demonstrated a lower shrinkage and a higher tensile strength. From the analysis of tensile fracture surfaces, it was observed that compared to the KH-550 modified and unmodified variants, the KH-570 modified nano-ettringite exhibits superior dispersibility in photocurable epoxy acrylate resin. Notably, when the amount of KH-570-modified nano-ettringite was 3 wt%, the highest tensile strength of the composite was 64.61 MPa, representing a 72.57% increase compared to the blank sample. Furthermore, the incorporation of KH-570-modified nano-ettringite as a filler provides a new perspective for improving the performance of photocurable epoxy acrylate resin composites.

## 1. Introduction

Photocurable resin materials include epoxy resin, phenolic resin, and acrylic resin, which offer major advantages through their ability to rapidly cure under ultraviolet (UV) radiation. However, resins are susceptible to warping and deformation during the process of light curing, generally resulting in low strength after curing. These two disadvantages of photocurable resin severely hinder the rapid development of light-curing three-dimensional (3D) printing devices in the industrial field [1,2]. To address these two drawbacks, materials with strengthening properties are added for the preparation of photocurable resin composites with directional properties. The incorporation of materials with strengthening properties can fully leverage the synergistic effect of two or more materials, thereby reducing the shrinkage and enhancing the tensile strength of photocurable resin composites [3,4,5].

As has been reported by numerous researchers, high-aspect-ratio materials possess a high specific modulus and specific strength, substantially enhancing mechanical strength when combined with photocurable resin to form composites [6,7,8]. He et al. [9] synthesized a composite material by adding glass fiber as the reinforcing phase to epoxy resin for light-curing rapid prototyping, demonstrating that increasing the glass fiber content in the system led to an improvement in the shear, bending, and tensile strengths of the resin. Liu et al. [10] coated calcium sulfate whiskers (CSWs) by using chitosan and subsequently modifying them with acryloyl chloride to form carbon–carbon double bonds on the surface. The modified CSWs were subsequently added to photosensitive resin. The results revealed that the incorporation of 5 wt% CSWs into the photosensitive resin increased both its tensile and impact strength by approximately 20% and 7%, respectively. Furthermore, the printed products exhibited high dimensional stability and accuracy.

Ettringite (3CaO·Al_2_O_3_·3CaSO_4_·32H_2_O), with a colorless or yellow hexagonal crystal, has garnered significant attention in the research and development of composite materials due to its high-aspect-ratio needle-like or fibrous morphology [11,12]. Compared to other high-aspect-ratio materials, ettringite offers lower raw material costs and simpler preparation processes [13]. Traditionally, it has been primarily used to enhance the performance of cement-based materials, such as improving compressive strength and reducing initial setting time, which contributes to its widespread application in construction engineering. Li Haiyan et al. [14] added 4 wt% ultrafine ettringite to a sulfoaluminate-cement-based material to effectively produce more high-aspect-ratio ettringite materials in cement hydration products compared with a control without ettringite. This approach led to an increase in compressive strength by 380% after 4 h and shortened the initial setting time by 55.6%. In recent years, with increasing demand for high-performance composite materials, the potential applications of ettringite are expanding into other fields [15,16]. In the domain of photocurable resin composites, the objective of incorporating ettringite is to enhance the strength and precision of the composite materials while preserving their lightweight characteristics. However, the use of ettringite in photocurable resin composites faces challenges such as achieving uniform dispersion and compatibility with the resin matrix. Investigating methods to improve the dispersion of ettringite within photocurable resins and enhance its interface bonding strength with the resin matrix is crucial for boosting the mechanical properties of these composites. 

In this study, high-aspect-ratio nano-ettringite was silanized using KH-550 and KH-570 to improve its dispersibility in epoxy acrylic resin. The silanized nano-ettringite was subsequently dispersed into epoxy acrylic resin to prepare resin composites. Moreover, in this study, the impact of silanized and untreated nano-ettringite on the viscosity, shrinkage, tensile strength, and elongation at break of photocurable resin composites was examined. These results can promote further exploration to expand the application range of high-aspect-ratio nano-ettringite as a reinforcing material.

## 2. Experiment

### 2.1. Materials

In this study, the materials utilized were γ-methacryloxypropyl trimethoxy silane (KH-570) and γ-Aminopropyltriethoxysilane (KH-550) produced by Sinopharm Chemical Reagent Co., Ltd. (Beijing, China); epoxy acrylic resin (4210) produced by Changzhou Ruisong Chemical Co., Ltd. (Changzhou, China); tripropylene glycol diacrylate (TPGDA) produced by New Land Polymer Materials Co., Ltd. (Zhaoqing, China); 2-hydroxy-2-methylpropiophenone (HMPP) produced by Kunshan Century Dragon Chemical Co., Ltd. (Suzhou, China); and nano-ettringite provided by the Nanomaterials Laboratory of Nanjing Tech University (Nanjing, China). 

### 2.2. Methodology

#### 2.2.1. Modification of Nano-Ettringite 

The following procedure was used to modify nano-ettringite: 1 g of KH-550 or KH-570 was added to 200 mL of ethanol solution (ethanol:water = 95:5), and the mixture was treated with ultrasound continuously for 30 min to completely hydrolyze KH-550 or KH-570. Then, 4 g of nano-ettringite was weighed out and added to the mixture. The mixed solution was stirred using a magnetic stirrer for 6 h in a water bath at 45 °C. Later, the solution was filtered using a vacuum pump, and the filter cake was washed repeatedly with anhydrous ethanol and dried at 40 °C for 12 h to obtain KH-550- or KH-570-modified nano-ettringite [17,18]. Finally, the modified nano-ettringite was finely ground using a mortar, after which the powder was sealed and stored.

#### 2.2.2. Preparation of Photocurable Resin Composites

**Figure 1:**
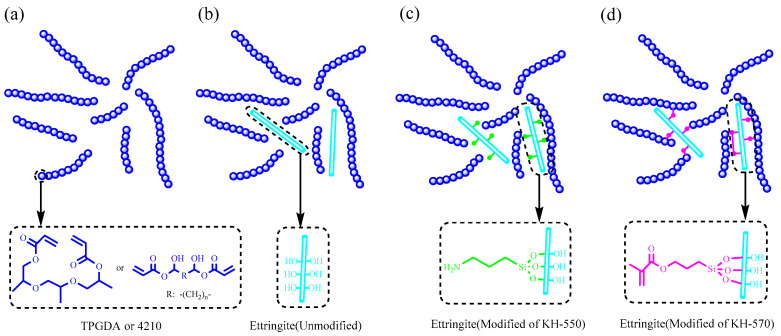
Crosslinking reaction of the pure resin (**a**); systems containing unmodified nano-ettringite (**b**); systems containing KH-550-modified nano-ettringite (**c**); and systems containing KH-570-modified nano-ettringite (**d**).

In this study, the resin precursor solution for light-curing 3D printing was obtained by mixing epoxy acrylate resin (4210) and an active monomer (TPGDA) at a molar ratio of 1:1 for 5 h. Then, 50 g of the resin precursor solution was weighed out, and 1 g of initiator was added along with modified or unmodified nano-ettringite in mass ratios of 0%, 3%, and 6%. The mixture was stirred while dispersing the other ingredients using ultrasound for 2 h to evenly distribute the modified or unmodified nano-ettringite in the mixed solution. The resulting evenly mixed solution was poured into a sample mold (a mold made of glass plates and coated with Vaseline in advance), cured under UV light for 10 s at a frequency of 40 kW using a light-curing device, and then removed to obtain the sample. The crosslinking system of photocured epoxy acrylate resin with modified and unmodified nano-ettringite can be observed in Figure 1.

### 2.3. Characterization

#### 2.3.1. Scanning Electron Microscopy (SEM) Analysis

A morphological analysis of the modified or unmodified nano-ettringite samples was conducted using a Zeiss Ultra55 (Carl Zeiss AG, Jena, Germany). For the analysis, high-vacuum mode was selected, the acceleration voltage was set to 5 kV, and the sample chamber pressure was set to 1 × 10^−3^ Pa. The sample preparation method is as follows: Take a small amount of dried modified or unmodified nano-ettringite powder with a toothpick, gently apply and spread it evenly on the double-sided carbon conductive adhesive on the sample holder. Use the toothpick to gently remove any excess nano-ettringite powder. Then, lightly press the surface of the powder sample with a clean glass slide. Use an ear bulb to gently blow away any powder not adhered to the conductive adhesive. Next, sputter a uniform gold film onto the surface of the powder sample, and then proceed with testing the sample. Additionally, the tensile fracture surface of modified or unmodified nano-ettringite/photocurable resin was characterized, and the morphology of the cross-section was examined.

#### 2.3.2. Fourier Transform Infrared (FTIR) Spectroscopy Analysis

To identify the differences in the nano-ettringite before and after modification, an FTIR spectrometer (Nicolet 8700, Bruker Optik GmbH, Ettlingen, Germany) with a wavenumber range of 4000–400 cm^−1^, applied in 32 scans, and a resolution of 4 cm^−1^ was utilized. The background spectrum was collected using a blank KBr plate.

#### 2.3.3. X-ray Diffraction (XRD) Analysis

The dried modified or unmodified nano-ettringite sample was uniformly coated onto a glass sample plate. Subsequently, phase analysis of the nano-ettringite sample was conducted using an X-ray (SmartLab, Rigaku, Tokyo, Japan) diffractometer with a diffraction step of 0.5°/min, and the adopted radiation source generated Cu-Kα X-rays at 40 mA and 40 kV.

#### 2.3.4. Thermogravimetric (TG) Analysis

Under an atmosphere consisting of 20% oxygen and 80% nitrogen, the nano-ettringite samples were characterized before and after modification using a TG analyzer (TG 409PC, NETZSCH Instruments Co., Ltd., Bavaria, Germany) at a heating rate of 10 °C/min in a temperature range of 30–800 °C.

#### 2.3.5. Viscosity Test

The liquid photopolymer resin containing modified and unmodified nano-ettringite was placed in a water bath at a temperature of 25 °C and subjected to 30 min of ultrasonic treatment and stirring. Throughout this process, a thermometer was used to ensure the liquid temperature stabilized at 25 °C. Subsequently, the viscosity of photocurable resin liquids with varying modified or unmodified nano-ettringite contents was tested using an NDJ-79 rotary viscosimeter following the GB/T 17473.5-2008 standard [19,20].

#### 2.3.6. Density and Shrinkage Testing

The volumetric shrinkage was measured according to the method described in reference [21]. The densities of both the photocurable resin liquids (*ρ_uncured_*) with various modified or unmodified nano-ettringite proportions and the solidified samples (*ρ_cured_*) were measured using an AR-120GY solid–liquid dual-purpose densimeter. The volumetric shrinkage (∆*V*) was calculated using the following equation:

$$\Delta V=(1-\frac{\rho_{\mathrm{uncured}}}{\rho_{\mathrm{cured}}})\times 100\%$$

#### 2.3.7. Testing of Mechanical Properties 

The mechanical properties, particularly the tensile properties and elongation at break of the modified or unmodified nano-ettringite/photocurable resin composites, were evaluated using a GX-9006 universal testing machine, following the GBT1040.3-2006 standard [22,23].

At this stage, resin was injected into the standard sample mold with a length of 115 mm and a width of 25 mm, as specified in GBT1040.3-2006, to obtain the samples. The fixture spacing was adjusted to 25 mm, and the stretching rate was set to 50 mm/min. The tensile strength (MPa) and elongation at break were determined as per the following formulas:

(1) Tensile strength: $\sigma_{t}=\frac{P}{b\cdot h}$

*b*—Sample width, mm.
*h*—Sample thickness, mm.
*P*—Breaking or maximum load, N.

(2) Elongation at break: $\varepsilon_{t}=\frac{\Delta L_{b}}{L_{0}}\times 100\%$

*L_0_*—Gauge length, mm.
△*L_b_*—Amount of elongation within the gauge distance *L*_0_ at the moment of sample fracture, given in mm.

## 3. Results and Discussion

### 3.1. SEM Analysis

**Figure 2:**
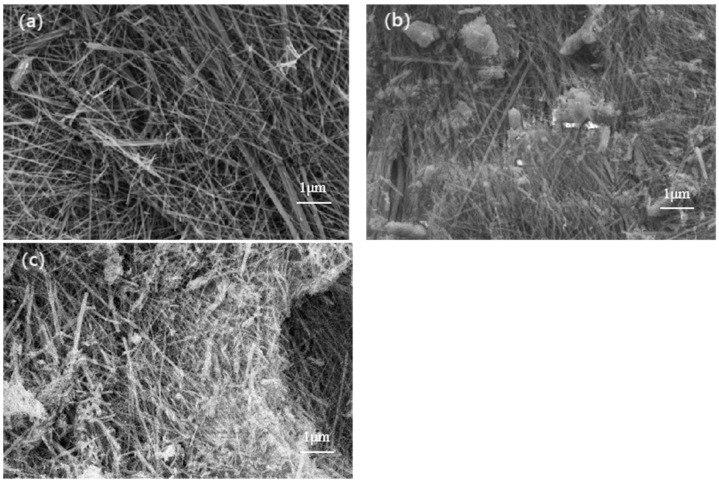
SEM images of unmodified nano-ettringite (**a**); KH-550-modified nano-ettringite (**b**); and KH-570-modified nano-ettringite (**c**).

In this study, the morphology and size of unmodified, KH-550-modified, and KH-570-modified nano-ettringite were observed using electron microscopy, as depicted in Figure 2. The evidence revealed by Figure 2a illustrates the microstructural clusters of unmodified nano-ettringite. These clusters are composed of densely packed nano-ettringite fibers, each measuring approximately 60 nanometers in diameter and over 10 μm in length. In contrast to the smooth crystal surfaces of the unmodified nano-ettringite, the nano-ettringite samples modified with KH-550 and KH-570 exhibit a plethora of fine particles covering their surfaces, as is evident from Figure 2b,c. This phenomenon can be attributed to two main factors: firstly, during the grinding process, the nano-ettringite crystals undergo fragmentation; secondly, the abundant adsorption of KH-550 and KH-570 via silane groups onto the surface of nano-ettringite induces a rougher surface morphology. Moreover, modification with KH-570 appears to be more effective than that with KH-550, as there is a greater coverage of fine particles on the surface of the nano-ettringite. Some researchers have pointed out that nanomaterials with high aspect ratios, after surface modification, develop three-dimensional rough structures that facilitate better bonding with resin matrices and enhance interfacial shear strength [24]. Hence, the modification of nano-ettringite using KH-550 and KH-570 could potentially enhance the ability of nano-ettringite to adhere to the resin matrix.

### 3.2. Infrared Analysis

**Figure 3:**
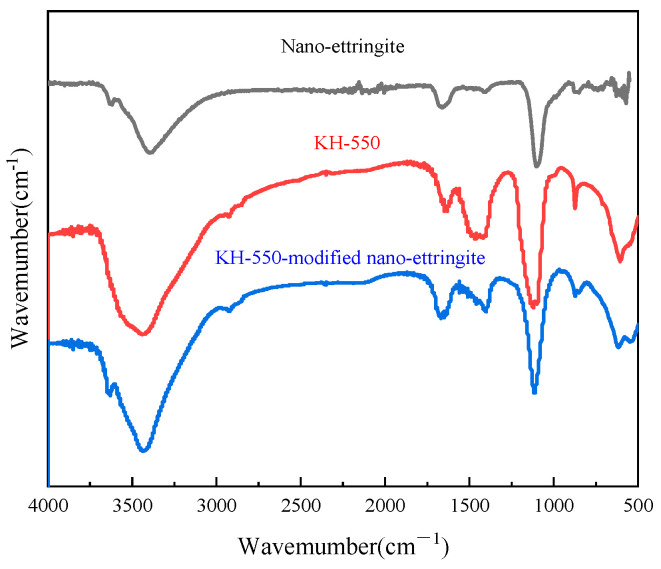
The infrared spectra of unmodified nano-ettringite, KH-550, and KH-550-modified nano-ettringite.

**Figure 4:**
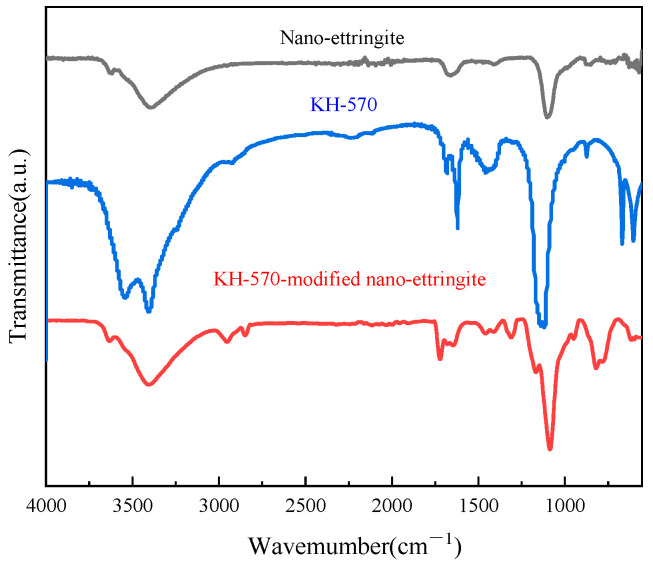
The infrared spectra of unmodified nano-ettringite, KH-570, and KH-570-modified nano-ettringite.

In this study, the silane coupling agents KH-550 and KH-570 employed were allowed to undergo Si/OH group reactions on the surface of the nano-ettringite through hydrolysis to enhance the interfacial adhesion between the nano-inorganic fillers and organic matrices, facilitate the dispersion of the nano-inorganic fillers in the organic photocurable resin, and leverage their unique size advantage [25]. Figure 3 illustrates the infrared spectra of the unmodified nano-ettringite, KH-550, and KH-550-modified nano-ettringite samples. The FTIR spectrum of nano-ettringite showed a broad peak at 3427 cm^−1^ due to the stretching vibrations of the -OH group on the surface of nano-ettringite. KH550 exhibits a broad absorption band at 3419 cm^−1^, attributed to the -OH and -NH_2_ functional groups. The peaks at 2928 and 890 cm^−1^ can be assigned to a symmetric methylene stretch (-CH_2_) and Si-O, respectively. These peaks also appear in the spectrum of the nano-ettringite modified with KH-550, indicating successful grafting of the coupling agent KH-550 onto the surface of the nano-ettringite. Figure 4 displays the infrared spectra of the unmodified nano-ettringite, KH-570, and KH-570-modified nano-ettringite. Notably, the infrared spectrum of KH-570 revealed a -CH_2_- characteristic peak near 2880 cm^−1^, a -C=O characteristic peak at 1730 cm^−1^, and a -Si-O characteristic peak at 890 cm^−1^. The peaks corresponding to -CH_2_-, -C=O, and -Si-O are all present in the spectrum of the KH-570-modified nano-ettringite, indicating the successful grafting of KH-570 onto the surface of nano-ettringite [18,26].

### 3.3. XRD Analysis

**Figure 5:**
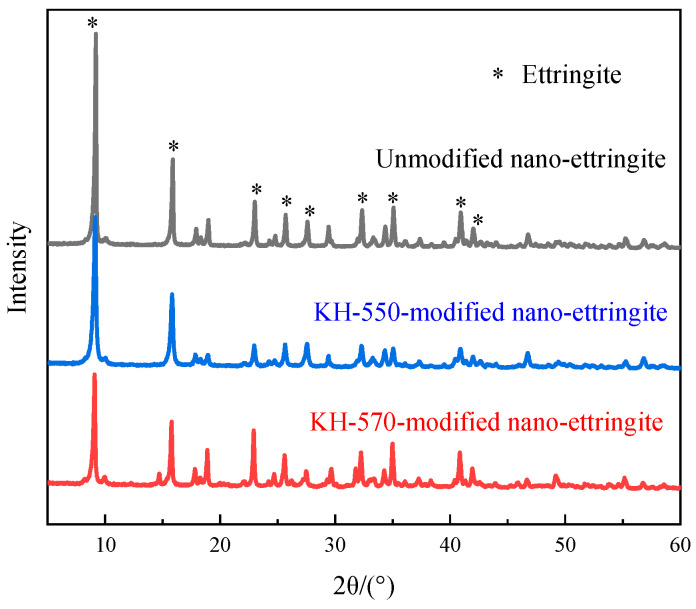
XRD patterns of unmodified, KH-550-modified, and KH-570-modified nano-ettringite.

Figure 5 presents the XRD analysis results for unmodified nano-ettringite, as well as nano-ettringite modified with KH-550 and KH-570 silane coupling agents. Upon comparing with the JCPDS data file for ettringite (Ca_6_Al_2_(SO_4_)_3_(OH)_12_·26H_2_O, PDF#41-1451), characteristic diffraction peaks of ettringite are clearly observed at 8°, 16°, 23°, 25°, 27°, 33°, 35°, 41°, and 43°2θ in all three spectra [27,28]. This consistent presence of peaks indicates that the crystal structure of nano-ettringite remains robust across all samples, unaffected by the modification process with either KH-550 or KH-570. The absence of new diffraction peaks in the modified nano-ettringite spectra confirms that the additions of KH-550 or KH-570 did not induce structural changes in ettringite. This outcome suggests that the silane coupling agents acted to encapsulate or modify the surface of nano-ettringite without altering its fundamental crystalline form. These findings are critically important for the incorporation of KH-550- and KH-570-modified nano-ettringite into UV-curable resins, as they maintain the integrity of its crystal structure and do not affect its original physical properties.

### 3.4. TG Analysis

**Figure 6:**
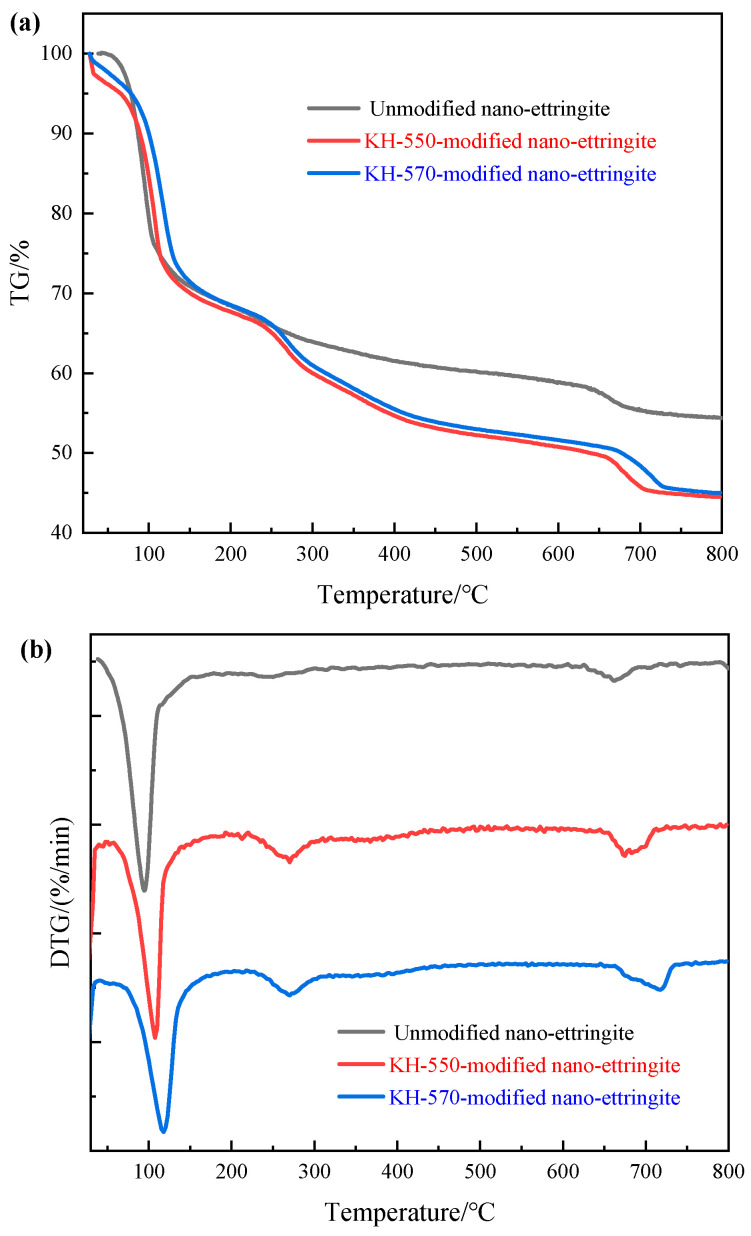
TG (**a**) and DTG (**b**) analysis graphs of unmodified, KH-550-modified, and KH-570-modified nano-ettringite.

The unmodified, KH-550-modified, and KH-570-modified nano-ettringite samples were heated from 30 to 800 °C at a heating rate of 10 °C/min in a nitrogen atmosphere to further verify the modification effects of KH-550 and KH-570. Subsequently, the grafting effect of KH-550 and KH-570 on nano-ettringite was analyzed by comparing the thermal weight loss rates of the samples [29]. As is depicted in Figure 6a, the thermal weight loss of the unmodified nano-ettringite sample increased upon increasing the temperature from 50 °C to 100 °C, following a linear trend, which is consistent with the observations made by Shuqiong Luo et al. [30]. The heightened increase in weight loss can be ascribed to the desorption of surface-adsorbed water on the nano-ettringite samples and the depletion of interstitial water within the aggregated nano-ettringite structures. Simultaneously, it can be observed that the thermal weight loss of nano-ettringite modified with KH-550 and KH-570 exhibits a linear trend as the combustion temperature increases from 50 °C to 100 °C, akin to unmodified nano-ettringite. Upon continuous heating, unmodified nano-ettringite exhibited three weight loss peaks at 94 °C, 242 °C, and 661 °C, as shown in Figure 6b and Table 1, attributed to significant structural water loss [31]. Post-modification with KH-550 and KH-570, these peaks were notably shifted to higher temperatures. Specifically, after KH-550 modification, the weight loss peaks of nano-ettringite were delayed to 108 °C, 262 °C, and 675 °C. With KH-570 modification, these peaks were delayed to 117 °C, 270 °C, and 717 °C. This indicates that modification with KH-550 and KH-570 significantly enhances the thermal stability of nano-ettringite. Additionally, the residual ratios of the KH-550-modified and KH-570-modified nano-ettringite samples after combustion were 44.48% and 45.00%, lower than the 54.40% of the unmodified nano-ettringite. This disparity may arise from the KH-550 and KH-570 coupling agent’s presence on the nano-ettringite surface. During combustion, the chemical bonds of KH-550 and KH-570 gradually deteriorate, decomposing into CH_4_ and CO_2_, and ultimately evaporating. The unmodified nano-ettringite samples, even after combustion at 800 °C, still retain certain aluminum oxide structures, which constitute high-temperature-stable compounds that are resistant to volatilization [32]. Consequently, this fact contributes to the relatively lower residual ratios observed in the KH-550- and KH-570-modified nano-ettringite samples.

### 3.5. Effect of Nano-Ettringite Dosage on the Viscosity of Photocurable Resin Composite Solutions

It has been observed that the viscosity of photocurable resin impacts the speed of 3D printing and the accuracy of devices [33]. Hence, it is imperative to analyze the viscosity changes of composite resins. Table 2 illustrates the impact of unmodified, KH-550-modified, and KH-570-modified nano-ettringite dosages on the viscosity of the analyzed photocurable composite resin. Notably, the viscosity of the composite resin increased as the amount of unmodified, KH-550-modified, and KH-570-modified nano-ettringite increased. This phenomenon can be attributed to the tendency of the higher-aspect-ratio nano-ettringite particles to overlap with each other, forming an internal skeleton system [34]. Consequently, it becomes difficult for the photocurable resin to move, resulting in increased viscosity [35]. At the same dosage, the viscosities of the KH-550-modified and KH-570-modified nano-ettringite composite resins were observed to be higher than those of the unmodified nano-ettringite composite resin This occurred because the surface of the nano-ettringite was modified with KH-550 and KH-570 coupling agents, which enhanced the interface adhesion with light-cured resin. What is particularly noteworthy is that, at equivalent dosages, the viscosity of the resin composite containing KH-570-modified nano-ettringite was higher than that of the resin containing KH-550-modified nano-ettringite. This result can primarily be attributed to the presence of double bonds in the structure of the KH-570 coupling agent, bonds that, under the influence of photoinitiators, readily undergo reactions with the double bonds in oligomer 4210 and the reactive diluent TPGDA, leading to the formation of larger molecules. Therefore, the enhanced adhesion between KH-570-modified nano-ettringite and the resin interface results in more difficult resin movement [36]. Overall, the viscosity of the composite resin system was below 700 mPa·s, satisfying the molding requirements for light-curing 3D printing.

### 3.6. Effect of Nano-Ettringite Dosage on the Shrinkage of Photocurable Resin

The shrinkage of photocurable resin is considered a major factor impacting 3D printing accuracy. It has been observed that during the curing of liquid resin, the van der Waals force distance between liquid molecules transitions to the covalent bond distance between solid polymer structural units, and the basic unit distance of molecules decreases, resulting in shrinkage during curing [37,38]. In this study, the photocurable resin was filled with inorganic non-shrinking components of unmodified, KH-550-modified, and KH-570-modified nano-ettringite to compensate for the gaps caused by the shrinkage of the organic components. Table 3 presents the impact of the dosage of unmodified, KH-550-modified, and KH-570-modified nano-ettringite on the shrinkage of photocurable resin. Notably, the curing shrinkage of the photocurable resin composite was reduced by the addition of unmodified or modified nano-ettringite; the higher the dosage, the smaller the degree of curing shrinkage. Furthermore, with the same dosage, the addition of KH-550-modified and KH-570-modified nano-ettringite resulted in a greater improvement in the curing shrinkage of photocurable resin compared to the addition of unmodified nano-ettringite. This is due to the addition of organic groups on the surface of nano-ettringite modified with the coupling agents KH-550 and KH-570, thereby enhancing the bonding ability at its interface with the photocurable resin and reducing the interface gap of shrinkage polymerization for the resin molecules [39]. With equivalent dosages, the nano-ettringite modified with KH570 exhibited lower shrinkage rates in composite materials with photocurable resin compared to those for the nano-ettringite modified with KH-550. This phenomenon can be attributed to the presence of double bonds in KH570, enhancing adhesion to the resin interface and resulting in fewer voids at the interface. Furthermore, this result confirmed that adding modified nano-ettringite to photocurable resin is more conducive to improving the accuracy of 3D-printing materials.

### 3.7. Effect of Nano-Ettringite Dosage on Tensile Strength and Elongation at Break of Photocurable Resin Composites

**Figure 7:**
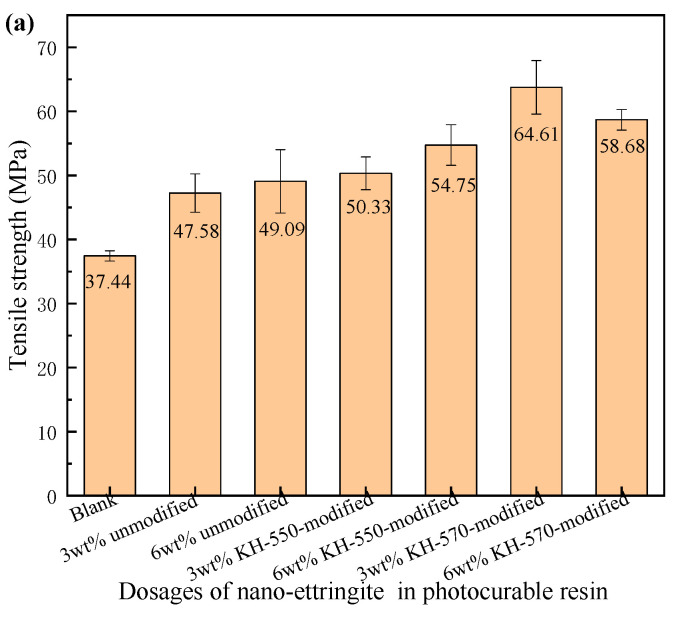

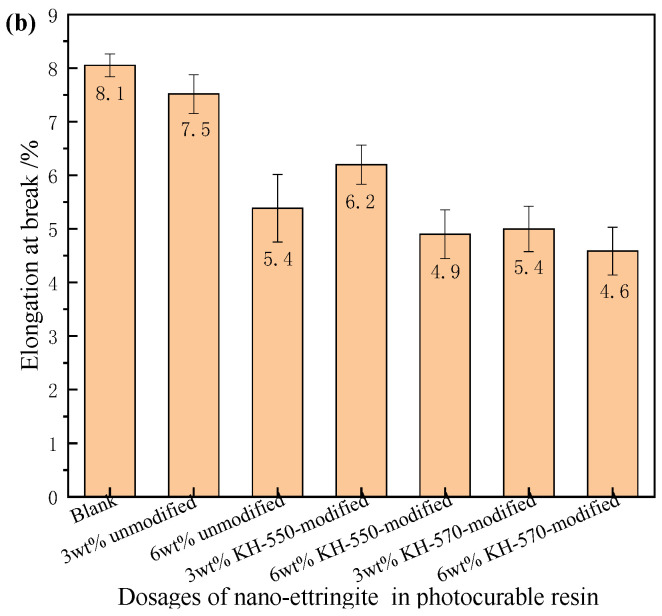
Effect of unmodified, KH-550-modified, and KH-570-modified nano-ettringite dosages on the tensile strength (**a**) and elongation at break (**b**) of photocurable resin composites.

Figure 7 depicts the tensile strength and elongation at break of photocurable resin composites containing varied amounts of unmodified, KH-550-modified, and KH-570-modified nano-ettringite. As is shown in Figure 7a, the tensile strength of the unmodified and KH-550-modified nano-ettringite samples increased with an increase in dosage. Meanwhile, the tensile strength of the KH-570-modified nano-ettringite samples initially increased and subsequently decreased with increasing dosage, though it remained higher than that of the blank sample. Compared to that of the unmodified nano-ettringite/resin composite, the tensile strength of the KH-570-modified nano-ettringite/resin composite increased by 17.03 MPa and 9.59 MPa, at the same dosages of 3 wt% and 6 wt%, respectively. This is indicative of the greater tensile strength the KH-570-modified nano-ettringite provides to the photocurable resin composite at a smaller dosage. However, agglomeration can occur when the dosage of KH-570-modified nano-ettringite is increased to a certain level, weakening its bonding strength with photocurable resin and resulting in reduced tensile strength [40,41]. Furthermore, the elongation at break of the samples decreased with an increase in the amount of unmodified, KH-550-modified, and KH-570-modified nano-ettringite incorporated (as shown in Figure 7b). Furthermore, the elongation at break of the samples containing KH-570-modified nano-ettringite was lower under the same dosage. This is because high-aspect-ratio nano-ettringite can easily form a crosslinked network structure, increasing its rigidity and reducing its elongation at break. Another reason for this is the stronger bonding ability at the KH-570-modified nano-ettringite and photocurable resin interface [42].

### 3.8. Tensile Fracture Surface Analysis

**Figure 8:**
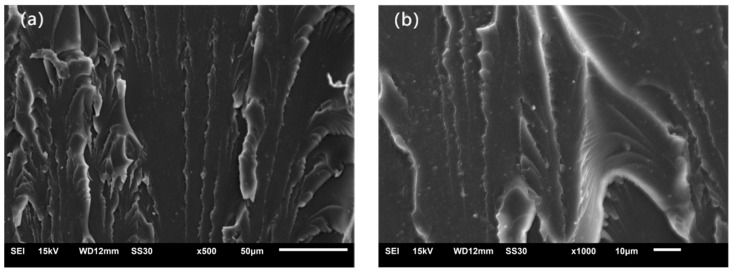

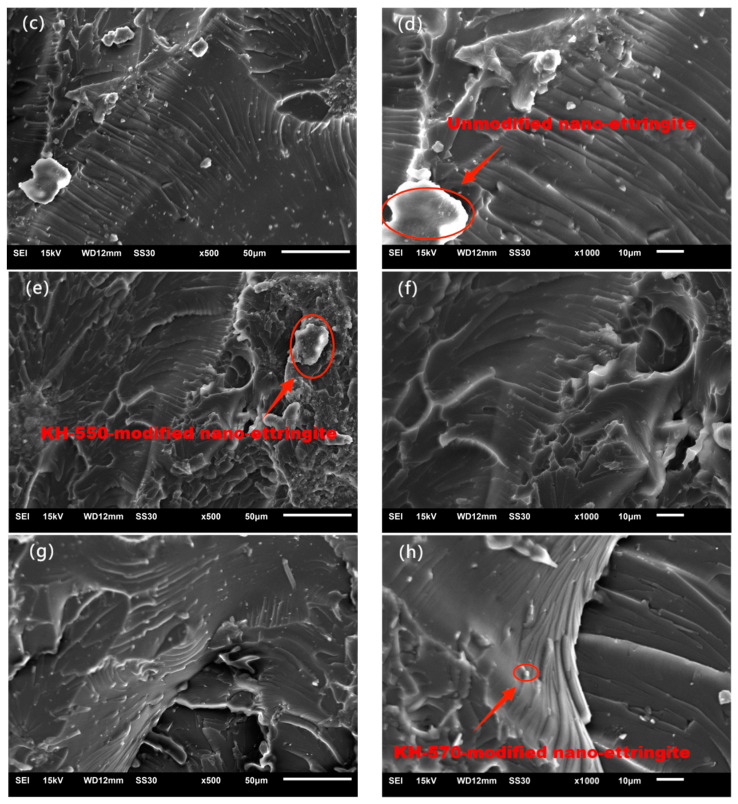
SEM images of the tensile fracture surfaces of the photocurable resin. (**a**,**b**) Pure resin; (**c**,**d**) 3 wt% unmodified nano-ettringite/resin composite; (**e**,**f**) 3 wt% KH-550-modified nano-ettringite/resin composite; (**g**,**h**) 3 wt% KH-570-modified nano-ettringite/resin composite.

Figure 8 provides the SEM images of the tensile fracture surfaces of pure photocurable resin and resin composites containing 3 wt% unmodified, KH-550-modified, and KH-570-modified nano-ettringite. As is depicted in Figure 8a,b, the tensile fracture surface of pure photocurable resin is relatively smooth without severe uneven tearing patterns, exhibiting typical brittle fracture behavior. Figure 8c,d displays numerous nano-ettringite clusters, indicating the poor dispersion of the unmodified nano-ettringite in the photocurable resin, which can easily cause stress concentration points and lead to an inability to fully utilize the advantages of nanomaterials [43]. In the SEM images of the tensile fracture surfaces in Figure 8e,f, it can be observed that nano-ettringite clusters are present in the resin matrix, and a significant portion of the resin matrix does not contain nano-ettringite, indicating that even after KH-550 modification, the compatibility between nano-ettringite and photocurable resin remains poor. Consequently, stress concentration phenomena persist. Figure 8g,h exhibit the even distribution of nano-ettringite in the photocurable resin, confirming that the nano-ettringite formed a good contact interface with the photocurable resin after being modified by the KH-570 coupling agent. This result indicates that compared to unmodified and KH-550-modified nano-ettringite, nano-ettringite modified with KH-570 exhibits better compatibility with photocurable resin. Consequently, their bonding was closer, which is conducive to the dispersion of KH-570-modified nano-ettringite in the resin matrix, thereby avoiding the generation of stress concentration points. Furthermore, the KH-570-modified nano-ettringite and photocurable resin demonstrated good compatibility, as evidenced by the close connection at the interface.

## 4. Conclusions

In summary, we modified nano-ettringite with KH-550 and KH-570, and analyzed the grafting effects using SEM, FTIR spectroscopy, XRD, and TG analysis. Furthermore, we conducted comprehensive research on the variations in viscosity, shrinkage, tensile strength, and elongation at break of unmodified, KH-550-modified, and KH-570-modified nano-ettringite when integrated into photocurable resin materials. Additionally, we analyzed the tensile fracture surfaces using electron microscopy. This investigation offers valuable insights into enhancing the compatibility between nano-ettringite and photocurable resin, thereby improving the application performance of photocurable resin composites. The research findings are outlined below.

1. SEM, FTIR spectroscopy, XRD, and TG analyses revealed that the surface of KH-550-modified and KH-570-modified nano-ettringite underwent successful siloxane grafting while retaining the original crystal structure of ettringite. Furthermore, the KH-550- and KH-570-modified nano-ettringite demonstrated enhanced thermal stability compared to unmodified nano-ettringite at elevated temperatures. The untreated nano-ettringite exhibited three weight loss peaks at 94 °C, 242 °C, and 661 °C, while after modification with KH-550, these peaks were observed at higher temperatures of 108 °C, 262 °C, and 675 °C. With KH-570 modification, these peaks were further delayed to 117 °C, 270 °C, and 717 °C.
2. As the dosages of unmodified, KH-550-modified, and KH-570-modified nano-ettringite increased, the viscosity of the photocurable resin composite rose, while the shrinkage diminished. At equivalent dosages, the KH-570-modified nano-ettringite exhibited higher viscosity but lower shrinkage compared to the unmodified and KH-550-modified nano-ettringite. The viscosity of resin composite materials with 3% by weight of unmodified and KH-550 modified nano-ettringite is 540 mPa·s. Upon modification with KH-570, the viscosity of the light-cured resin composite material increases to 560 mPa·s. Additionally, the shrinkage rate of resin composite materials containing 3% by weight of unmodified nano-ettringite is 8.01%. After modification with KH-550 and KH-570, the shrinkage rates of the light-cured resin composite materials decrease to 7.97% and 7.92%, respectively.
3. Under identical dosages, the photocurable resin composite incorporating KH-570-modified nano-ettringite demonstrated greater tensile strength and reduced elongation at break compared to the unmodified and KH-550-modified nano-ettringite samples. The optimal dosage of KH-570-modified nano-ettringite was observed at 3 wt%, achieving the highest tensile strength of 64.61 MPa, in the photocurable resin composite, representing a 72.57% increase compared to that of the blank sample. However, the fracture elongation at this loading was 5.4%, which is lower than the blank sample’s 8.1%.
4. Analysis of the tensile fracture surfaces showed that the unmodified and KH-550-modified nano-ettringite displayed numerous clusters within the photocurable resin. Conversely, the KH-570-modified nano-ettringite exhibited uniform dispersion, indicating its superior compatibility with the photocurable resin.

## Figures and Tables

**Table 1:** TG and DTG analysis data of unmodified, KH-550-modified, and KH-570-modified nano-ettringite.

Nano-Ettringite Sample	Initial Weight/mg	Residue Weight/mg	Residual Ratio/%	T_1_/°C	T_2_/°C	T_3_/°C
Unmodified	15.89	7.25	54.40	94	242	661
KH-550-modified	13.76	7.64	44.48	108	262	675
KH-570-modified	35.72	19.65	45.00	117	270	717

Note: T_1_ is the temperature of the first thermal weight loss peak, T_2_ is the temperature of the second thermal weight loss peak, and T_3_ is the temperature of the third thermal weight loss peak.

**Table 2:** Effect of unmodified, KH-550-modified, and KH-570-modified nano-ettringite dosages on the viscosity of photocurable resin composites.

Sample	Viscosity (mPa·s)
Pure resin	420
3 wt% unmodified nano-ettringite/resin composite	540
6 wt% unmodified nano-ettringite/resin composite	610
3 wt% KH-550-modified nano-ettringite/resin composite	540
6 wt% KH-550-modified nano-ettringite/resin composite	620
3 wt% KH-570-modified nano-ettringite/resin composite	560
6 wt% KH-570-modified nano-ettringite/resin composite	640

**Table 3:** Effect of unmodified, KH-550-modified, and KH-570-modified nano-ettringite dosages on the shrinkage of photocurable resin composites.

Sample	Liquid Density (g/cm^3^)	Solid Density (g/cm^3^)	Shrinkage/%
Pure resin	1.1030	1.2050	8.46
3 wt% unmodified nano-ettringite/resin composite	1.1140	1.2110	8.01
6 wt% unmodified nano-ettringite/resin composite	1.1305	1.2242	7.65
3 wt% KH-550-modified nano-ettringite/resin composite	1.1156	1.2122	7.97
6 wt% KH-550-modified nano-ettringite/resin composite	1.1301	1.2234	7.63
3 wt% KH-570-modified nano-ettringite/resin composite	1.1188	1.2150	7.92
6 wt% KH-570-modified nano-ettringite/resin composite	1.1289	1.2219	7.61

## Data Availability

Data are contained within this article.
